# Stem Cells in Dentistry: Types of Intra- and Extraoral Tissue-Derived Stem Cells and Clinical Applications

**DOI:** 10.1155/2018/4313610

**Published:** 2018-07-02

**Authors:** Ana Gomes Paz, Hassan Maghaireh, Francesco Guido Mangano

**Affiliations:** ^1^Department of Endodontics, Lisbon, Dental School, University of Lisbon, Lisbon, Portugal; ^2^Clinical Teaching Fellow, University of Manchester, Manchester, UK; ^3^Department of Medicine and Surgery, Dental School, University of Varese, Varese, Italy

## Abstract

Stem cells are undifferentiated cells, capable of renewing themselves, with the capacity to produce different cell types to regenerate missing tissues and treat diseases. Oral facial tissues have been identified as a source and therapeutic target for stem cells with clinical interest in dentistry. This narrative review report targets on the several extraoral- and intraoral-derived stem cells that can be applied in dentistry. In addition, stem cell origins are suggested in what concerns their ability to differentiate as well as their particular distinguishing quality of convenience and immunomodulatory for regenerative dentistry. The development of bioengineered teeth to replace the patient's missing teeth was also possible because of stem cell technologies. This review will also focus our attention on the clinical application of stem cells in dentistry. In recent years, a variety of articles reported the advantages of stem cell-based procedures in regenerative treatments. The regeneration of lost oral tissue is the target of stem cell research. Owing to the fact that bone imperfections that ensue after tooth loss can result in further bone loss which limit the success of dental implants and prosthodontic therapies, the rehabilitation of alveolar ridge height is prosthodontists' principal interest. The development of bioengineered teeth to replace the patient's missing teeth was also possible because of stem cell technologies. In addition, a “dental stem cell banking” is available for regenerative treatments in the future. The main features of stem cells in the future of dentistry should be understood by clinicians.

## 1. Introduction

Stem cells are undifferentiated cells, capable of renewing themselves. Via differentiation, they have the potential to develop into many different cell lineages. There are different kinds of stem cells, depending on the type of cells they can create and the location in the body. In recent years, studies have shown that oral tissues are a source of stem cells. Structuring of tissue in dentistry has revealed promising results in the regeneration of oral tissue or organs. There are multiple factors that can produce alveolar bone resorption due to tooth extraction or loss because of severe cavities, trauma, or root fracture or even because of periodontal diseases. In edentulous patients, bone resorption continues throughout life particularly in the mandible, which makes it difficult to substitute the missing teeth with dental implants [1].

Tissue engineering therapies and stem cells are a promising way to achieve alveolar bone regeneration and solve large periodontal tissue defects and finally to substitute a lost tooth itself. Organs and tissues such as tongue, salivary glands, the temporomandibular joint condylar cartilage, and skeletal muscles are set to be used in regenerative dental medicine.

To develop the concept of oral tissue and organ regeneration for clinical application in dentistry, several studies have been carried out in animals including key elements of tissue engineering such as extracellular matrix scaffolds and stem cells [2]. Furthermore, clinical trials about jaw bone regeneration applied in dental areas such as implantology using stem cells and tissue engineering strategies have demonstrated positive results.

Considering the new role of regenerative biology and stem cells in dentistry, especially regarding the ideal stem cells for oral regeneration, some confusion can be made depending on the various oral and maxillofacial locations where stem cells can be obtained [3].

The aim of this review is to explain the different kinds and sources of stem cells from a clinical perspective in dentistry, regarding their accessibility, immunomodulatory properties, and differentiation capacity, as well as their clinical applications. We will focus on the ongoing analysis and clinical studies in dentistry.

## 2. Origins

### 2.1. Pluripotent Stem Cells

The pluripotent stem cells when applied in dentistry can include investigation on the biology and regenerative treatments due to their pluripotency and unlimited self-renewal. Dental research is focused on obtaining oral lineages from the differentiation of pluripotent stem cells to be applied clinically [4].

#### 2.1.1. ES Cells (Embryonic Stem Cells)

ES cells are produced from the culturing cells, which precede from the blastocyst, particularly from its undifferentiated inner cell mass (the early stage of embryonic development after fertilization) [5]. They are of great interest because of their particular distinguishing quality of differentiating in vitro into all somatic cell lineages and germ cells [6]. The main reason why there are moral and ethical questions about the use of human ES cells has to do with the embryonic origin.

Research about pluripotent stem cells and its differentiation may help to understand the oral developmental biology and in future can be useful to create strategies in regenerative dentistry to fulfill the clinical demands [7]. Nevertheless, these kinds of studies are expensive, and researchers still have to deal with ethical issues, unless experts, who can routinely deal with patient embryos, were included in the team.

#### 2.1.2. iPS Cells (Pluripotent Stem Cells)

iPS cells have the aptitude to develop into various types tissue and organs. This stem cell technology is very promising, which can revolutionize medicine and create a biocompatible medicine that uses patients' cells to supply individual and biocompatible treatments.

IPS cells can be obtained from multiple oral mesenchymal cells: SCAP, DPSCs and SHED, TGPCs, buccal mucosa fibroblasts, gingiva fibroblasts, and periodontal ligament fibroblasts [8]. It is expected that oral cells can be an ideal iPS cell source, which can be applied in regenerative procedures for periodontal tissue, salivary glands, missing jaw bone, and tooth loss [9].

iPSCs are obtained by introducing reprogramming factors or specific products of pluripotency-associated genes into a given cell type. The original set of reprogramming factors are the transcription factors Oct4 (encoded by the gene POU5F1), Sox2 (sex-determining region Y-box 2), cMyc, and Klf4 (Kruppel-like factor 4). Each of these factors can be replaced by related transcription factors, miRNAs, small molecules, or even nonrelated genes such as lineage specifiers [10].

Duan et al. described that making the combination between iPS cells and enamel matrix derivatives can enhance periodontal regeneration and the cementum formation of the periodontal ligament and alveolar bone [11]. Other studies suggested that the ability of iPS cells to differentiate into ameloblasts and odontogenic mesenchymal cells is promising in tooth bioengineering [9, 12].

Further research is necessary to understand how to control their differentiation. It is still unclear whether iPS and ES cells are equal.

It is necessary to identify iPS cell origins to achieve adequate guided differentiation. Furthermore, if iPS cells are clinically applied, it is important to prevent tumor formation upon in vivo implantation, since its protocol of implantation uses the oncogene c-Myc, which can raise concerns about possible carcinogenic properties. However, this problem can be solved by using L-Myc replacing c-Myc and reprogramming using components which are not viral, such as proteins, microRNA, synthetic mRNA, or episomal plasmids. Nevertheless, remaining undifferentiated iPS cells that stay among the differentiated target cells can uncontrollably proliferate to form teratomas in the transplanted location, being an important clinical problem. To solve this concern, different methods such as a cell sorting approach or a selective ablation procedure have been investigated [1].

### 2.2. Adult Stem Cells

Embryonic stem (ES) and adult stem cells are two of the leading sources of stem cells present in humans. Further sources can be obtained synthetically from somatic cells, which are known as pluripotent stem (iPS) cells.

Adult stem cells can only develop into a certain number of kinds of cells. On the other hand, ES cells or IPS cells are pluripotent stem cells, which means that they can differentiate into all kinds of cells from all three germinal layers.

There are very few adult stem cells existing in adult tissues that go through self-regeneration and differentiation to maintain healthy tissue and repair damage tissues. They are known to be somatic stem cells or postnatal stem cells [13, 14] that undergo into self-renewal and differentiation to repair injured tissues. Studies on stem cells have revealed that there are in the oral and maxillofacial location a number of adult stem cell sources [15].

#### 2.2.1. Introduction to MSCs

Even though bone marrow was the original source of MSCs, there are alternatives which have been drawn from other adult tissues [16–18]. Thanks to their capacity of self-renewing and their ability to differentiate along specific lines on stimulation, these types of cells present promising characteristics for the development of cell-based approaches in bone regeneration [17].

Friedenstein et al. described in the 70s the approach of using adherent fibroblastic cells that were drawn from the bone marrow [19] and their capacity to differentiate into several mesenchymal tissues. Years later, Pittenger et al. described human mesenchymal stem cells from the iliac crest bone marrow as multipotent cells, explaining their isolation, expansion in culture, and differentiation into chondrogenic, adipogenic, and osteogenic lineages [20]. Nevertheless, due to the lack of homogeneity of the population of bone marrow isolated adherent cells and the inability to identify definitive markers for MSCs, this concept of MSCs is still controversial [21]. Mesenchymal stem cells can be attached to tissue culture-treated plastic when maintained in standard culture conditions [22] as stated in ISCT criteria. In addition, MSCs should express CD105, CD73, and CD90 and lack the expression of CD45, CD34, CD14 or CD11b, CD79a or CD19, and HLA-DR surface molecules. In vitro, MSCs must also be able to differentiate into chondroblasts, adipocytes, and osteoblasts [23]. In 2007, studies have identified other cell surface markers for human MSCs like CD271 [24] and MSC antigen-1 [25]. Finally, the selection of MSC's fixed mRNA markers shown in MSCs [26, 27] has been reported.

#### 2.2.2. Bone Marrow-Derived MSCs (BMSCs)

BMSCs are multipotent progenitor cells present in adult bone marrow. Due to their replicative capacity, they can also differentiate into numerous cells of the connective tissue. BMSCs can be isolated from the iliac crest [28].

Many studies have demonstrated that BMSCs from the iliac crest can differentiate into myogenic, osteogenic, chondrogenic, adipogenic, and nonmesenchymal neurogenic lineages [29]. Even though the process of isolating BMSCs from the bone marrow is a relatively simple process, a major surgical and invasive operation will be needed, and this is considered one of the great drawbacks of BMSCs from the iliac crest. Nevertheless, this procedure is the most common and it has been used in dental bone regeneration for many years.

Thanks to the high potential for regenerating bone [30], BMSCs from the human iliac crest are important for bone tissue engineering notwithstanding patient age [31, 32].

Still, various reports have described a relation between age and the reduction in the osteogenic potential of BMSCs when extracted from the femur and iliac crest [32–34] and delineate that the age of the donator is an important factor for bone formation. Furthermore, the expansion capacity seems to be restricted, since cells tend to age and lose their properties with repeated passaging and culture time in their multidifferentiation potential. The disadvantages must be overcome to apply with success BMSCs for bone regeneration and tissue engineering.

We can obtain BMSCs from orofacial bones as well. Human BMSCs can be isolated from the maxilla and mandible bone marrow suctioned during dental treatments like dental implantation, third molar extraction, orthodontic osteotomy, or cyst extirpation [35].

These cells have the possibility to be attained from both young patients (6–53 years old [36]) and from older patients (57–62 years old [36]), taking into consideration that the age of the donor can have some influence on the gene expression pattern of BMSC [37].

Animal [37–39] and human studies [40–42] have described that grafted bone from the craniofacial area for autologous bone grafting at craniofacial locations produces greater results and considerably higher bone volume than bone extracted from the edochondral bone, such as rib or iliac crest.

Depending on the BMSC niche and type present in the graft, distinct skeletal different skeletal tissues have distinguishing regenerative qualities.

Following embryology, cranial neural crest cells create maxilla and mandible bones, and the mesoderm originates the iliac crest bone. This embryological explanation may be the reason why there are functional differences between the iliac crest human and orofacial BMSCs [41–44].

Studies revealed that orofacial BMSCs have functional and phenotype differences compared to the iliac crest BMSCs. In 2007, a group of researchers described that BMSCs derived from the orofacial site have a reduced differentiation potential with distinct expression patterns for several MSC marker genes when compared to the ones derived from the ilium, femur, and tibia [26]. Authors like Akintoye et al. reported specific site properties of the BMSCs derived from the orofacial and iliac crest of the same individual, where a greater proliferation and osteogenic differentiation ability was observed from the BMSCs derived from the orofacial site compared to the ones from the iliac crest. Furthermore, orofacial BMSCs' adipogenic potential is lower than those of the iliac, [43] which can lower the production of fat during bone tissue regeneration. The properties described from the orofacial BMSCs can be considered advantageous for bone regeneration. Nevertheless, the volume collected from the iliac crest bone marrow is higher than that from the orofacial bone marrow (0.03–0.5 ml) [36–45]. To sum up, authors suggest that, when applying BMSCs in clinical trials, a safe cell expansion and more reliable protocol must be rooted.

#### 2.2.3. Dental Tissue-Derived Stem Cells

Epithelial stem cells and MSC-like cells have been described in dental tissues. In 1999, through organ culture of the apical end of the mouse incisor, the first epithelial stem cell niche was established. The cervical loop of the tooth apex where the niche is located possibly contains dental epithelial stem cells, which have the ability to turn into enamel-producing amelobasts. There is no information available about human dental epithelial stem cells. This niche can be particular to rodents, since their incisors are different from all human dentition, erupting continuously throughout the animal's life.

Having the suitable conditions after dental procedures, dental tissues such as dental pulp and periodontal tissues are able to regenerate and form reparative dentine. We can find mesenchymal progenitor or stem cells in these types of tissues [46].

Various sources of MSC were verified in dental tissues, and isolated stem cells were also studied [47].

#### 2.2.4. Periosteum-Derived Stem/Progenitor Cells

Periosteum is the name given to the specialized connective tissue whose function is to cover the outer surface of the bone tissue. In 1932, author Fell firstly described the osteogenic potential of long bones periosteum and its membrane, having suggested its capacity to form a mineralized extracellular matrix if there were the suitable in vitro circumstances [48]. The histological periosteum composition is based on 2 different tears and up to 5 very distinct functional locations when dissociated enzymatically and cultured [49]. The external area contains elastic fibers and fibroblasts, and the interior area is constituted by MSCs, fibroblasts and osteoblasts, osteogenic progenitor cells, microvessels, and sympathetic nerves [50].

These cells have the ability to differentiate into adipocytes, osteoblasts, and chondrocytes and to express the typical MSC markers. Furthermore, it was described that single-cell-derived clonal populations of adult human periosteal cells have a multipotential mesenchymal property, since they can turn into adipocytes, chondrocytes, osteoblasts, and skeletal myocyte lineages in vivo and in vitro. This can explain why periosteum-derived cells could be used in tissue engineering, in particular for bone regeneration.

Clinical research has demonstrated positive results when cells derived from the periosteum were applied for sinus or alveolar ridge augmentation, which showed reliable implant insertion, with improved bone remodeling and lamellar bone production, and also demonstrated that shorter postoperative waiting time was needed after implantation.

As a result, in case of large bone defects, the periosteum could be a source of stem/progenitor cells [51].

#### 2.2.5. Salivary Gland-Derived Stem Cells

Salivary gland-derived stem cells have been studied to be used for autologous transplantation treatment, for gland tissue engineering, and for cell treatments. The endoderm originates from the salivary glands, which compose the epithelial cells from the ductus and acinar cells with exocrine capacity. The epithelium proliferates when the link of the salivary gland duct occurs, and the acinar cells undergo apoptosis.

Stem cells that can differentiate into all kinds of epithelial cells within the gland have not yet been identified in literature [52, 53]. Salivary gland stem/progenitor cells were isolated from a rat submandibular gland, and it was found that these cells are highly proliferative and have the ability to express acinar, myoepithelial, and ductal cell lineage markers [54].

Studies suggest that salivary glands are a promising source for stem cells that can be used for therapy in patients that suffer from cancer to the head and neck and who have undergone radiotherapy.

Human salivary gland primitive MSC-like cells were isolated that evidence embryonic and adult stem cell markers and can be guided to differentiate into chondrogenic, osteogenic, and adipogenic cells [55]. The selection of a cell's particular marker or label with induced reporter proteins is essential to obtaining a considered actual stem cell culture for the salivary gland [56].

#### 2.2.6. Adipose Tissue-Derived Stem Cells (ASCs)

Adipose tissue has been studied as a stem cell source in regenerative medicine, and it is considered an abundant MSC source. ASCs can be obtained through lipectomy or from lipoaspiration from areas such as the chin, hips, upper arms, and abdomen with low donor-site morbidity, as liposuction is a very common cosmetic procedure [57].

ASCs are expected to be an alternative source of MSCS in bone regeneration in the dental field, as they present a robust osteogenesis [58].

The practicability of using ASCs in GBR and implant surgery has already been tested [59].

More studies are needed, focusing on ASCs to be used clinically with efficacy in periodontal and bone regeneration.

### 2.3. Regenerative Dentistry with Stem Cell Application

A suitable stem cell must carry out the control of cell outcome, guaranteeing patient safety in regenerative medicine.

MSCs currently have been described to have a clinical potential, since their regeneration potential in bone and periodontal tissue has been evaluated, and there are some clinical studies already published.

#### 2.3.1. Differentiation Capacity

BMSCs, especially periosteum-derived stem cells or bone marrow-derived stem cells, are appropriate for alveolar bone growth due to their compatibility with the target tissue. MSCs can also present promising results for dental mesenchymal-derived tissue regeneration, like periodontal tissues, pulp, or dentin. Nevertheless, MSCs' capacity of differentiating is restricted to mesenchymal lineages, which can retard the regeneration of complex oral organ application, since they are formed during development by epithelial and mesenchymal tissue interaction.

An option to achieve organ regeneration is to identify specific organ stem cells based on the ability of a single tissue-specific stem cell to form gastric units or epithelial components of the mammary glands [60, 61].

Studies have already demonstrated that pluripotent stem cells are a solution for complex organ renewal [62, 63], since there are no postnatal stem cells with organogenic capacity in teeth or salivary glands. Nevertheless, it is necessary to understand how it leads iPS cells to achieve specific progenitor cells for the tissue and organs targeted for renewal to obtain successful results. Further studies based on the development of iPS cell technology are necessary.

#### 2.3.2. Immunomodulation

Immunomodulation has been identified in MSCs with therapeutic effects in angiogenesis, anti-inflammation, and antiapoptosis. Studies also described that MSCs have a short inherent immunogenicity [64]. Other studies described that MSCs derived from human oral tissue (SHED, PDLSCs, SCAP, and GMSCs) have immunomodulatory characteristics equal to BMSCs [65–68].

Gingiva can be considered a promising origin of stem cells with future potential for immune-related therapies as well as for regenerative medicine, since GMSCs promote the oral mucosa progenitor cells to have a fetal phenotype with immunomodulation to be recognized by our immune system [69].

#### 2.3.3. Regeneration

MSCs hold promise in regenerative therapies due to their multipotency and availability. MSCs are being considered for the treatment of a wide range of pathologies, and researchers are especially interested in their potential to treat musculoskeletal disorders such as osteoarthritis, osteoporosis, and osteonecrosis [70].

An important MSC application in dentistry is pulp and dentin regeneration. Cell-based approaches in endodontic regeneration based on pulpal MSCs have demonstrated promising results in terms of pulp-dentin regeneration in vivo through autologous transplantation. Despite that pulpal regeneration requires the cell-based approach, several challenges in clinical translation must be overcome including aging-associated phenotypic changes in pulpal MSCs, availability of tissue sources, and safety and regulation involved with expansion of MSCs in laboratories. Allotransplantation of MSCs can be an alternative in going through these obstacles; more research needs to be carried out on the long-term stability of MSCs and efficacy in pulp-dentin regeneration [71].

## 3. Clinical Applications

### 3.1. Evolution in Regenerative Therapy in Dentistry

Stem cell action contributes as a main factor to the capacity of self-renewal and differentiation of every organ and tissue.

The regeneration of lost oral tissue is the target of stem cell research. Owing to the fact that bone imperfections [72] that ensue after tooth loss can result in further bone loss which limits the success of dental implants and prosthodontic therapies, the rehabilitation of alveolar ridge height is prosthodontists' principal interest.

There are already different regenerative therapies based on stem cell technology available, namely, mesenchymal stem/stromal cells (MSCs). Although these cells have already been used in the clinic for alveolar bone augmentation, hardly anything is known about their in vivo biology [73].

In the clinic, the main approach to the treatment was the material-based reconstruction without major surgical procedures; nonetheless, the clinical concept was expanded, including stem cell-based regeneration, as a consequence of the emerging stem cell technologies and the requirements of alveolar ridge augmentation associated with implant dentistry [73].

The development of bioengineered teeth to replace the patient's missing teeth was also possible because of stem cell technologies.

The approach of regenerative dentistry has already been applied in implantology and periodontology [74]. In this text, we are going to do an assessment of the progress in regenerative therapies associated to periodontal tissue and alveolar bone.

#### 3.1.1. Tissue Regeneration Based on Scaffolds

The periodontal regenerative therapy concept is based on the principal that, firstly, the source of infection must be removed and, secondly, a space for the cells to grow must be provided [75]. Guided tissue regeneration (GTR) is the most documented material used in periodontal regeneration [76, 77]. In this kind of regeneration, biocompatible barrier membranes are used to cover the bone defects. Using this technique, connective tissue and bone regeneration occurs within the bone defect. The bone defect is protected by a barrier with migration of epithelial tissues into the wound [78]. Bioinert materials, such as pure titanium membranes, PLGA, and ePTFE, cannot stimulate bone formation [79]. GBR and socket preservation are alveolar bone augmentation and preservation techniques that demand the application of bioactive materials to raise the activity of bone formation and therefore provide direct bonding with the bone. Hydroxyapatite, tricalcium phosphate (b-TCP: OSferion 1, Olympus, Japan), biphasic calcium phosphate, and bovine bone mineral are CaP-based biomaterials. These materials are not osteoinductive materials since they cannot stimulate production of new bone in locations with lack of bone [80]. To permit and speed up bone formation and augment osteointegration of implants (underrating implant failure), the osteoinduction using bone grafting substitutes can be a solution when titanium implants are applied. For that reason, osteoinductive scaffolds based on CaP were engineered through osteogenic bioactive factor incorporation and have been reported to stimulate bone formation [81, 82].

Due to the fact that natural extracellular matrix (ECM) components modulate MSC osteogenic differentiation, adhesion, migration, and proliferation, it is beneficial for scaffolds to mimic the ECM [83].

Nevertheless, due to safety issues, it is not possible to apply them in the clinic animal-derived ECM. Other encouraging alternatives are synthetic peptide analogues of ECM components or bioactive small molecules [84].

For ECM-based biomimetic material acquisition, cell-derived decellularized extracellular matrices are likely to yield favorable results [85].

#### 3.1.2. Growth Factor Delivery-Based Tissue Regeneration

Approaches which combine with scaffold-based tissue regeneration options have been increased by the growth factor delivery [86, 87]. The usage of platelet-rich plasma (PRP) is a well-known therapy which applies growth factor release to obtain periodontal regeneration. PRP can be utilized to regenerate periodontal defects, since it contains a variety of matrix components and growth factors. To obtain predictable periodontal regeneration, there is high interest in considering the application of PRP in combination with bone grafts or autologous stem cells [88].

A recent innovation in the field of medicine and dentistry is the development of autologous platelet-rich fibrin (PRF) as a growth factor delivery system. PRF is a platelet concentrate next to platelet-rich plasma with an advantage of simplified preparation and no biochemical blood handling. PRF represents a new step in the platelet gel therapeutic concept with simplified processing without artificial biochemical modification. The combined properties of fibrin, platelets, leucocytes, growth factors, and cytokines make platelet-rich fibrin a healing biomaterial with tremendous potential for bone and soft tissue regeneration. Interestingly, in 2014, a new protocol for PRF was introduced (termed Advanced-PRF or A-PRF) whereby centrifugal forces were decreased and total spin times were increased. This modification to centrifugation protocol has previously been shown to increase platelet cell number and monocyte/macrophage behavior [89].

Differences in growth factor components and platelet count between different PRP preparation procedures may be the reason why there are inconclusive results of clinical trials of PRP [90]. Enamel matrix derivative (EMD) product has also been extensively applied in periodontology for regeneration procedures [90, 91].

Some studies already described that EMD inhibits epithelial cell growth and induce periodontal fibroblast growth which may help in periodontal tissue regeneration [90–92].

Recombinant growth factors such as PDGF-BB and FGF-2 and BMP-2 were introduced for bone and periodontal regenerative treatments [93]. BMPs are known for their ability to induce bone formation and for playing an important role in embryonic patterning and early skeletal formation.

Another major factor in platelet-rich plasma is PDGF, which is known to induce angiogenesis [94, 95].

FGF-2 is a growth factor delivery, as it has several biological functions in tissue regeneration, inducing formation and growth of blood vessels and stem cell proliferation [93, 96].

MSC cultures are reported to stimulate bone formation in rats [97].

### 3.2. Stem Cells' Regenerative Therapy Requirements

#### 3.2.1. Augmentation of Alveolar Bone

Taking into consideration that regular bone grafting materials have no osteoinductive properties, it is difficult to accomplish through material/growth factor-based procedures such as bone augmentation of the acutely atrophic alveolar ridge, especially vertical bone augmentation during guided bone regeneration or sinus-lifting. Activated osteoclasts bring out an unavoidable resorption which is the immune response against the transplants; even when used in combination with scaffolds, host cells are not able to migrate into a large defect area.

Due to the fact that autologous cancellous bone contains osteogenic, osteoconductive, and osteoinductive features provided by a suitable cellular content, it has been applied for big bone defects [98]. Nevertheless, the limited intraoral supply and difficulty in harvesting for autologous grafts have inspired another alternative method: the development of stem cell-based tissue engineering treatment [99]. Since the increasing demand of dental implants, there has also been an increasing demand for techniques related to bone augmentation in atrophic alveolar ridge and maxillary sinus.

Stem cells present an encouraging strategy to accomplish the regeneration of large alveolar bone defects, accelerate bone formation, and stimulate osteointegration in implant treatments.

### 3.3. Treatments Based on Stem Cells

The clinical application of stem cells has been analyzed in cases of alveolar ridge augmentation in dental implant rehabilitation. The clinical applications of stem cell-based bone augmentation are split into two groups: the chair-side cellular grafting and the tissue engineering approach. In either case, the most frequently applied stem cells are BMSCs from the iliac crest [100].

#### 3.3.1. Approach of Tissue Engineering

The regenerative strategies using stem cells have utilized cell culture techniques to achieve bone tissue engineering [101].

Dental pulp-derived MSCs in combination with a collagen sponge scaffold can be used to restore human mandible bone defects. Regardless of the fact that stem cell-based tissue engineering has been suggested to be beneficial, there is criticism on the absence of characterization of the cellular component of the graft which can foreseeably produce consistent cell populations [102].

It is necessary to verify if tissue engineering based on cells ultimately has advantages for patients and to decide definitive protocols for stem/osteoprogenitor cell preparation. Further studies on this subject are needed.

#### 3.3.2. Approach of Chair-Side Cellular Grafting

Cellular graft derived from patients and prepared clinically or an allograft bone matrix that contains native MSCs is another alternative of bone regeneration based on stem cells [103].

There is evidence and good documentation about cellular grafting methods applying the mononuclear fraction obtained from processed fresh marrow. One of these methods is called “bone marrow aspirate concentrate (BMAC).” Stem cells that have the function of hematopoiesis and MSC population are two of the principal lineages of stem cells present in the mononuclear fraction [104].

The cells in freshly processed grafts may contain a variety of cell types, that is, stromal cells, angiogenic cells, MSCs, osteogenic cells, and hematopoietic cells. Some studies have reported that when BMSCs are administrated to an injured tissue or intravenously, it can have a positive anti-inflammatory effect [105, 106].

Further studies and research are needed to explain in detail the precise mechanisms of implanting BMSC population.

#### 3.3.3. Tissue Regeneration Based on Cell Sheet

Cell sheet-based tissue regeneration has been applied successfully in tissue regeneration [107–110]. Enzymatic cell digestion and cell-to-cell contact are not needed since they remain intact, which is an advantage for regeneration of tissue. In addition, ECM proteins can be applied without requiring an additional scaffold.

A variety of cell sheets in tissue engineering have been described, for instance, using the cell sheet as a source of 3D pellet, applying multilayered cell sheet, and using the cell sheet to wrap a scaffold [111–116].

This technology has already been applied in periodontal and alveolar bone tissue regeneration [117–119].

Researchers reported that dental follicle cells (DFCs) could be an alternative for root and periodontal regeneration [120].

### 3.4. Regenerative Therapy Based on Stem Cells: Influencing Factors

The therapy based on stem cells is a new technology that has shown promising results for orofacial bone regeneration; nevertheless, these procedures are still poorly understood.

More clinical evidence is needed to understand if the new bone that was formed was provided by the implanted cells which survived or is from host osteogenic cells [121].

#### 3.4.1. Transplanted Cells' Survival

Osteogenic cells which have the ability to retain the cellular activity to allow the cells that are transplanted to be able to produce ECMs for tissue regeneration are required for tissue engineering to be successful through cell transplantation [120].

Nevertheless, the destiny of cells and their clinical results are still unknown.

It was observed in animal studies that the cells that are transplanted can migrate out of the transplanted location or die quickly [122, 123].

In 2010, Tasso et al. demonstrated in an animal study that distinct waves of cells (CD31^+^ endothelial progenitors and CD146^+^ pericyte-like cells) migrated from the host to the BMSC-seeded ceramic to develop new tissue [124].

One year later, Boukhechba et al. proved that BMSC cells which were grafted did not survive more than a month after they were implanted [125].

More studies are needed to help understand the interaction of cells in bone regeneration treatment.

#### 3.4.2. Donor Cells: The Preculture Condition

The preculture condition of cells that are transplanted was widely analyzed on bone formation.

It has been suggested that human BMSCs lose their in vivo osteogenic capacity in in vitro expansion, when cultured not regarding the osteogenic induction length [126].

Preculture periosteum-derived cells with biomimetic calcium and phosphate supplementation resulted in partial or complete ectopic bone formation, although CaP-based biomaterials have significant potential for bone regeneration [127].

The period is an important factor of in vitro preculture to regenerate the bone using BMSCs.

In majority of cases, undifferentiated MSCs were found; nevertheless, osteogenically induced MSCs were solely found in fewer cases. Thus, we can conclude that the host immune system can destroy these.

Optimal conditions for human BMSCs should be established once the protocol for bone regeneration based on stem cells is designed.

#### 3.4.3. Cellular Grafting: Local Immune Responses

Ectopic bone formation applying stem cells that are transplanted in animal models does not have clinically predictable results for orthotopic bone formation in individuals.

The donor BMSCs can produce several anti-inflammatory factors to restrict the capacities of the various types of immune cells [128]. Even though the results of MSC-mediated immunosuppression are a restriction of T cell activation and proliferation, MSCs have also been shown to induce T cell differentiation into immunosuppressive Tregs [129, 130]. Furthermore, MSCs provoke recipient T cell apoptosis, resulting in an augmentation in the number of Tregs [131]. MSCs may also stimulate dendritic cells and macrophages to secrete IL, which in turn has an immunosuppressive effect on T cells [132].

Future clinical applications will be guided by BMSC biology, environment, and interactions.

### 3.5. Complex Oral Tissue/Organ Regeneration: Preclinical Studies

Due to their developmental and structural complexity, it was not possible to do a clinical trial about regeneration technologies for complex oral organs and tissues on the head and neck. Nevertheless, there are some advances based on animal research that have been known as good strategies to regenerate these tissues.

#### 3.5.1. Root/Tooth Regeneration

The aim of tooth regeneration is to obtain a functional tooth which can replace the lost one [133]. Root regeneration is now a more clinical applicable approach. Studies reported that using the root/periodontal complex constructed using periodontal and apical papilla stem cells would be able to support an artificial crown to provide normal tooth function in a model of a swine [134]. Additionally, DFCs were successfully used for tooth root reconstruction together with dentin matrix scaffold.

Tooth regeneration is one of the most important achievements in dentistry. Tooth structures from mice, rats, and pigs have been used in tooth engineering [135].

Bioengineered tooth transplantation has been proven to be a solution for tooth regenerative treatments, especially when an important alveolar bone loss exists [136].

This procedure is still an obstacle clinically when using tooth regeneration technology, and iPS cells can be considered a cell source [12].

#### 3.5.2. Regeneration of Salivary Glands

Salivary gland regeneration is an interesting topic especially for head and neck oncology experts. Two regenerative approaches to restore the function of salivary glands have been applied. The first application is to obtain an artificial salivary gland by tissue engineering. The second application is to use stem cells in the damaged salivary tissue. There are some reports in specialized literature that refer that stem cells such as MSCs and BMSCS can be applied to reestablish the function of the damaged salivary glands [137].

A recent review article describes that using genetic lineage tracing in mice, the DNA label application to mark label-retaining quiescent cells, in vitro floating sphere assays, and two-dimensional (2D) or three-dimensional (3D) cultures of both human and rodent salivary glands cells demonstrated multiple stem/progenitor-like cells in the salivary glands. These cells can be identified and isolated, thanks to the expression of proteins and enzymes. These stem/progenitor cells present at different occasions during organ development and may compensate cell loss to allow suitable organ formation. Even during adult salivary gland homeostasis, multiple reservoir cell types in compartments have the ability to duplicate, maintain, and/or expand themselves [138].

#### 3.5.3. Regeneration of Mandible Condyle

Tissue regeneration can be a solution to temporomandibular joint disc condyle defects or trauma. El-Bialy et al. reported in their study that BMSCs could increasingly regenerate a rabbit condyle that was enhanced by using pulsed ultrasound [139]. All these findings can help develop the concept for stem cell-based tissue engineering if there is condyle degeneration in case of disorders like rheumatoid arthritis.

#### 3.5.4. Tongue Regeneration

Tongue regeneration has already been reported in animal studies with the objective of reconstructing tongue defects and reestablishing speech, swallowing function, and air protection [140, 141]. Cell-based reconstruction of the tongue was reported in a rat model, in which myoblast-progenitor cells were implanted in a hemiglossectomized tongue for muscle regeneration [140]. Nevertheless, functional regeneration is difficult in the tongue. In 2013, Egusa et al. reported in their study that the application of cyclic strain to BMSCs stimulates the achievement of aligned myotube structures [142]. More advanced studies in stem cell engineering may help develop the regenerative techniques of the damaged or resected tongue and reestablish its role [142].

### 3.6. Immunotherapy with MSCs

MSCs have been expanded for the therapy of immune diseases.

#### 3.6.1. Application BMSCs in Immune-Mediated Diseases

BMSCs constitute an important HSC niche component in the bone marrow [143].

They act in the repair process, thanks to cytokine and growth factors' secretion and endogenous progenitor cells' proliferation and differentiation [144]. Thus, transplanted or endogenous MSCs are stimulated by inflammatory cytokines (TNF-*α*) [145]. In addition, MSCs express matrix metalloproteinase to come through ECM barriers [146]. Some studies reported that BMSCs present an important immunomodulatory action. Therefore, it can be applied as a treatment for immune disorders [147, 148]. Thus, peripheral tolerance is induced by the administration of BMSCs, and the BMSCs then move to damaged tissues, where the release of proinflammatory cytokines is inhibited and cell survival is encouraged [148].

Several animal studies have examined BMSCs' effect in immune-mediated inflammatory diseases [149]. Additionally, MSCs' immunosuppressive effect in patients in case of refractory inflammatory bowel disease and graft versus host disease (GVHD) [150, 151] has been proven.

More studies are needed to explain MSCs' immune-modulatory effect before applying these cells therapeutically.

#### 3.6.2. Immunotherapy with MSCs in Dentistry: Possible Applications

Reports demonstrated that transplanted allogeneic PDLSC sheets show decreased immunogenicity and marked immunosuppressive ability [151].

Studies reported the systemic delivery of dental MSCs to be applied in therapeutic strategies, since they can curb Th17 cell differentiation and an augmentation in the number of Treg cells [66, 152, 153].

All new MSCs' immunomodulatory features may be interesting to dental experts since they can be used for regenerative therapy and immunotherapy.

### 3.7. Banking of Stem Cells in Dentistry

Specialized studies have demonstrated that dental tissues are a rich source of MSCs, which can be applied in medical fields, particularly in immune and regenerative therapies [154].

The process of storing stem cells acquired from patients' deciduous teeth and wisdom teeth, called dental stem cell banking, is a strategy to realize the potential of dental stem cell-based regenerative therapy [155].

Stem cell-containing tissues are acquired from the patient and can be cryopreserved for many years to retain their regenerative capacity. Whenever required, dental stem cells, which are tolerated by the immune system, can be isolated from the cryopreserved tissue/tooth for future regenerative therapies [156, 157].

## 4. Conclusions

The oral and maxillofacial regions have been described as a promising source of adult stem cells. Dental clinicians should recognize the evolution of the regenerative dentistry field and take into consideration the possibility of acquiring stem cells during dental treatments (from deciduous teeth, third molars, and the gingiva), which can be stored for future autologous therapeutics.

We obtain iPS cells from discarded oral tissues that can be used in patient-specific modeling of oral diseases and the development of tailor-made diagnostic and drug screening tools for alveolar bone augmentation and oral cancer treatment, apart from the autologous cell-based regeneration of complex oral tissues. Nevertheless, more studies are needed to justify the application of these cells in autologous regenerative cells in the dental field.

Further studies on adult MSCs and BMSCs are needed to identify factors that have the responsibility to achieve successful results of stem cell-based bone and periodontal tissue regeneration. It is also important that researchers investigate more about the immunomodulatory properties of the stem cells, thus facilitating the grafting of transplanted cells at inflamed sites.

Further studies on adult stem cells and pluripotent stem cells should be developed to obtain more effective outcomes in the regenerative dentistry field.

Since it has more predictable regenerative results, future research areas of stem cell-based therapy in dentistry should be focused on tissue engineering and chair-side cellular grafting approaches.

To achieve more scientific evidence, more studies, such as clinical randomized controlled trials with long follow-ups, must be carried out.

There must also be a complete understanding of biological processes on both donor and recipient sides during bone regeneration which is extremely important to be able to structure more effective clinical strategies for stem cell-based bone regeneration.

MSCs' immunomodulatory function is important in suppressing the local immune response during transplantation and in achieving optimal tissue regeneration.

Prosthodontists are being motivated to get involved in stem cell biology by the increased requirement for new technologies for implant dentistry.

Authorized organizations should establish a link between stem cell-based dentistry, with standard protocols, so it can more often be applied in the dental field.
